# Spectrophotometric Method for the Determination of Atmospheric Cr Pollution as a Factor to Accelerated Corrosion

**DOI:** 10.1155/2017/7154206

**Published:** 2017-04-02

**Authors:** Dereje Homa, Ermias Haile, Alemayehu P. Washe

**Affiliations:** Department of Chemistry, Hawassa University, P.O. Box 05, Hawassa, Ethiopia

## Abstract

The effect of Cr(VI) pollution on the corrosion rate of corrugated iron roof samples collected from tanning industry areas was investigated through simulated laboratory exposure and spectrophotometric detection of Cr(III) deposit as a product of the reaction. The total level of Cr detected in the samples ranged from 113.892 ± 0.17 ppm to 53.05 ± 0.243 ppm and showed increasing trend as sampling sites get closer to the tannery and in the direction of tannery effluent stream. The laboratory exposure of a newly manufactured material to a simulated condition showed a relatively faster corrosion rate in the presence of Cr(VI) with concomitant deposition of Cr(III) under pH control. A significant (*P* = 0.05) increase in the corrosion rate was also recorded when exposing scratched or stress cracked samples. A coupled redox process where Cr(VI) is reduced to a stable, immobile, and insoluble Cr(III) accompanying corrosion of the iron is proposed as a possible mechanism leading to the elevated deposition of the latter on the materials. In conclusion, the increased deposits of Cr detected in the corrugated iron roof samples collected from tanning industry zones suggested possible atmospheric Cr pollution as a factor to the accelerated corrosion of the materials.

## 1. Introduction

Accelerated corrosion of corrugated iron roof (galvanized-steel) is a subject of global concern because of its importance to the service life of the material and its aesthetic appearance [1, 2]. Atmospheric corrosion is the result of a redox reaction between the metal component of the material and its atmospheric environment that occurs in the presence of a conducting thin aqueous adlayer [3]. The common incorporation of pollutant species into this adlayer usually enhances the degradation process. Although steel structures are employed in atmospheric environments with some means of surface protection, considerable researches have demonstrated the effects of both steel alloy composition and atmospheric environments on its corrosion behavior [1]. For instance, the ability of zinc to galvanically protect iron is relatively effective in neutral environment but very sensitive to any change of atmospheric acidity [2]. Air pollutants such as sulfur dioxide, hydrogen sulphide, oxides of nitrogen, and chlorides and weathering factors such as temperature, moisture, rainfall, solar radiation, and wind velocity have been recognized as conventional atmospheric parameters that may contribute to the corrosion [1–3]. Industrial sites particularly those in most tropical locations are the most corrosive sites due to the polluting chemicals (such as H_2_S and SO_2_-precursors of acid rain), solid particles in the atmosphere including soot, the time of wetness (humidity), and the high temperature experienced [1, 2]. The synergistic interaction of the atmospheric pollution variables and coupled processes can also play considerable role in the corrosion phenomena. For instance, the presence of metallic pollutants such as Cr that can galvanically couple with the iron of the roofing material through the adlayer can accelerate the degradation process [4–7]. The major sources of Cr in the atmosphere are industries including leather tanning industries, textile (printing, dyeing), chromium plating, steel production, and refractories [8, 9]. Among these, the leather tanning industry is the major source of chromium in the environment due to the disposal of chromium-contaminated sludge [9–11]. Studies have already indicated the above regulation limit contamination of soil, water, and vegetables in villages adjacent to tanneries by Cr [12–14]. Although Cr(III) is the most expected form in the tannery effluents, the incidence of the hexavalent form can occur due to redox transformations occurring in the sludge [15–18]. Chromium may also present in the atmosphere in the form of particulates and aerosol droplets [19–21]. Chromium from sources releasing the element in lager particles (particle diameter varies 0.2–50 *µ*m) is deposited locally and can migrate through individual environmental media. Transport within the terrestrial and water systems is greatly affected by chemical speciation; chemical forms of chromium and their affinity to chemical and photochemical redox transformations; precipitation/dissolution and adsorption/desorption process, for example, occurring in individual compartments of the biogeochemical cycle of chromium [20]. Redox conversion of Cr^3+^ to Cr^6+^ can increase Cr^6+^ dislocation from the soil into the water systems and the atmosphere [20, 21]. Previous researchers have demonstrated that Cr(VI) is stable in oxidizing environment with pH above 6.0 but tends to reduce to more thermodynamically stable and insoluble Cr(OH)_3_ under conditions of pH 3 to 6 [22, 23]. The hexavalent chromium can also be reduced to the trivalent chromium compound through a coupled reaction that causes iron corrosion [4–6]. Reduction of Cr(VI) to Cr(III) by reaction with zerovalent iron (Fe^0^) and subsequent precipitation of Cr(III) oxyhydroxides can occur through the reaction sequence [4]:

(1) CrO4aq2−+Fes0+8Haq+⟶Feaq3++Craq3++4H2Ol1−xFeaq3++xCraq3++2H2Ol⟶Fe1−xCrxOOHs+3Haq+

**Scheme 1:**
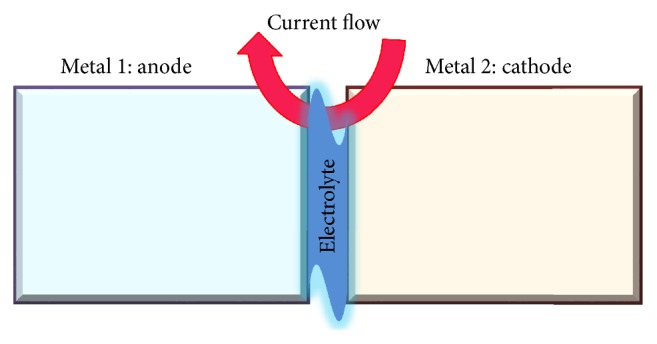
Electrochemical cell configuration for bimetallic corrosion.

Such coupled redox reactions are well known from bimetallic corrosion. Because the zerovalent iron and Cr(VI) have different natural reduction potentials, current will flow from the cathode Cr(VI) to the anode (iron) metal via the conducting liquid at the metallic junctions under moist conditions, which will increase the corrosion rate on the anode. Acidic moisture from precipitation, fog or dew, or other sources serves as an electrolyte in this type of atmospheric galvanic corrosion. Since there is an excess of oxygen in the atmosphere, the corrosion of iron sheet in atmospheric environment is not limited by the amount of oxygen present and can proceed rapidly when the electrolyte is present. The electrochemical cell configuration thus consists of one metal (more electropositive) as anode and the other as cathode (more electronegative) connected via the junction electrolyte as indicated in Scheme 1 [24].

Since the trivalent chromium compounds are thermodynamically stable, immobile, and sparingly soluble, it is envisaged that the level of Cr in the roofing material might increase (above the initial level in the alloyed material-steel) as a result of reduction of Cr(VI) to Cr(III) by reaction with Fe^0^ and subsequent precipitation of Cr(III). The objective of the current study is, therefore, to determine chromium deposits on roofing iron as both a factor and indicator for accelerated corrosion of the roofing iron sheets by chromium (VI) transformation in adjacent villages of the Akaki-Kaliti industrial zone of Ethiopia. Several analytical techniques including inductively coupled plasma-mass spectrometry (ICP-MS), inductively coupled plasma-atomic emission spectrometry (ICP-AES), electrochemical analysis, spectrophotometry, neutron activation analysis, and atomic absorption spectrophotometry (AAS) are available for the determination and speciation of Cr(III) and Cr(VI) either in off-line or in on-line methods [25, 26].

In this study off-line procedures are employed in the spectrophotometric determination and speciation of Cr(III) and Cr(VI) because of its simplicity. Spectrophotometric determination of Cr(VI) by complexation with 1,5-diphenylcarbazide was used as a quicker and easier method [27]. Thus AAS was used for the determination of total Cr and UV-Vis for Cr(VI). Speciation was carried out based on the difference of results from the two methods. A simulated laboratory exposure of a newly manufactured corrugated iron sample to synthetic corrosive air was also conducted under different conditions to study the transformation of Cr(VI) to Cr(III) and the accelerated corrosion of the iron material in the coupled galvanic process. Exposure of a metal to a corrosive atmosphere leads to the formation of corrosion products, which usually remain on the surface leading to mass gain. Laboratory exposure of metals to synthetic environments containing SO_2_ and/or NO_2_ with humidified air has been used by different research groups for the investigation of the atmospheric corrosion of metals and proved to be reliable [28–31].

## 2. Materials and Methods

### 2.1. Description of the Study Area and Study Design

The research was conducted at Akaki-Kaliti industrial zones of Ethiopia which is located to the south of Addis Ababa in the Akaki-Kaliti subcity. The subcity covers an area of 124.7 sq.km with a total population of 194,002, 2015 census. More than 200 industries including the Ethiopian Tannery Share Company (ETSC) have been registered in the subcity. Based on the interview made with households residing in the subcity and appropriate stakeholders including Addis Ababa Environmental Protection Authority, villages adjacent to the Ethiopian Tannery Share Company (ETSC) were selected as the target sites. The ETSC is located 85 km southeast of Addis Ababa with a grid reference of 8°27.154′ latitude and 39°03.894′ longitudes. This area is characterized by a semiarid climate having an altitude of 1630 m, an average annual rainfall of 800 mm, and minimum and maximum temperature of 17.5°C and 26°C, respectively. Samples of roofing iron were then systematically collected within a kilometer radius of effluent discharge point.

### 2.2. Samples Collection and Preparation

Three different groups of the roofing iron materials were collected. The first group consisted of samples collected from areas very close to tanning industry zones where a high level of Cr emissions was reported by previous researchers [12–14]. These samples were collected from two directions: along the direction of the tannery effluent discharge line (east corrugated) and opposite to the direction of the tannery effluent discharge line (west corrugated). Four sampling sites at 100 m intervals were considered in each case giving a total of eight samples. Only samples with the same years of service that appeared corrosively damaged were systematically selected. The second group of samples referred to as control groups were collected from presumably natural environment in rural areas that are about 200 km and farther from industrial sites. The third group of samples called a universal control consisted of the newly manufactured corrugated roofing iron purchased from four different shops. All the samples belonged to the same brand and manufacturer. The samples were cut into pieces with plastic nail and packaged in plastic bag containers and transported to the laboratory. The sampling was carried out in March 5 to 9/2016.

### 2.3. Instrument, Apparatus, and Chemicals

In this work Flame Atomic Absorption Spectrophotometer (Buck Scientific, Model 210VGP AAS, USA) equipped with deuterium background corrector and air-acetylene flame atomizer was used for determination of the total Cr in corrugated iron samples. Spectrophotometer (model UNICAM UV-300, England) was used for determination of Cr(VI). All the samples were weighed on a digital microbalance with a 0.0002 mg precision. Stock standard solutions (Buck Scientific calibration standards, USA) containing 1000 mg/L of total Cr metals were used. Instrumental parameters of the FAAS for determination of total Cr were adjusted according to the manufacturer recommendation. The correlation coefficient of the calibration curve for the metal was 0.9992 which assured the linearity of instrumental response for analytes. For Cr(VI) a blank and the standard solutions were analyzed with UV-Visible spectrophotometer with 1 cm quartz cell which was used and the absorbance measurements were performed in the range of 300–800 nm. The absorption maxima were observed at 540 nm and *r* = 0.998. 1,5-Diphenylcarbazide (BDH, India) complex and K_2_Cr_2_O_7_ were used for the preparation of standard solutions. Deionized water was used for all solution preparation solutions.

### 2.4. Sample Digestion and Preparation for FAAS

Digestion of iron samples was performed following the optimized conditions. Briefly, 1.00 g of the corrugated roofing iron was cut into small pieces, dissolved in 30 ml of 5 M HCl and 10 ml conc. HNO_3_, and heated in the solution at 75°C for 1 hr. Then the solution was filtered into a 100 ml volumetric flask and diluted with distilled water up to the mark. Each of the above iron samples was digested in triplicate.

### 2.5. Speciation through Determination of Cr(VI) by UV-Vis Spectrophotometer

An analytical grade of 250 mg of 1,5-diphenylcarbazide (DPC), a common complexing agent of chromium (VI) for spectrophotometric analysis, was taken and dissolved with acetone and diluted to 100 ml with distilled water. Then a freshly prepared 1 ml of DPC was added to each sample and aged until a red violet colour was developed. Analytical grade 0.5 g of K_2_Cr_2_O_7_ was taken and dissolved with deionized water and diluted to 500 ml. Then 10 ml of the standard was taken and further diluted to 100 ml. A series of ten standards containing 1, 0.5, 0.2, 0.04, and 0.01 mg were prepared and acidified with 0.2 N of 5 ml of sulfuric acid. Then a freshly prepared 1 ml of DPC was added to each standard and a pink colour was immediately developed. Then sample analysis was performed on the clear solutions obtained from the digestion procedure after filtration through Whatman number 45 for analysis by the 1,5-diphenylcarbazide method at 540 nm.

### 2.6. Laboratory Exposures

**Scheme 2:**
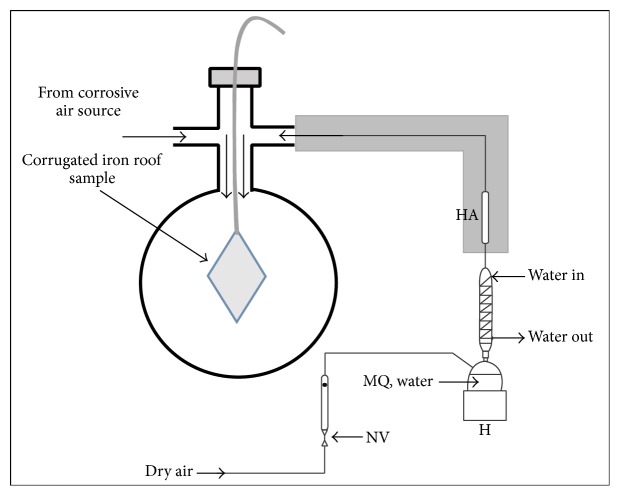
Schematic experimental set-up to exposure of corrugated iron roof to simulated atmospheric condition (HA = humid air; NV = valve).

To study the impact of Cr(VI) pollution on atmospheric corrosion of corrugated iron roof, an atmosphere with controlled gaseous corrosion simulators was used. A home built humidity chamber was employed where the synthetic corrosive air generated in a separate reactor and humidified air from opposite source was mixed in a column where the iron sample was held [32]. The corrugated iron sample was previously exposed to different conditions prior to exposure to the synthetic air. The exposure to acidic condition was carried out by submerging the sample in H_2_SO_4_ solutions of different pH (3, 5, 7, and 9) in presence and absence of Cr(VI) for 30 minutes to form a surface layer containing the required chemicals and then dried at 55–60°C and weighed before exposure to the corrosive air. Then the control samples (submerged in MQ water at pH = 7) and test samples of different wet surfaces were taken out, dried, weighed, and placed in the reaction chamber where they were further exposed to synthetic air. The synthetic air is composed of the humidified air and the corrosive air generated in separate sources and allowed to get mixed in the exposure chamber, where the iron sample was held. The container of MQ water was submerged in a thermostatic bath to maintain the temperature at 25 ± 0.5°C. A schematic description of the experimental set-up for exposure of corrugated iron roof to simulated atmospheric condition is shown in Scheme 2. The exposure chamber was constructed using a simple distillation flask with double side arms, an ambient air circulator, and septum as a covering lid. The ambient air enters from above through a ball valve and gets mixed with lateral flows of humidified and synthetic corrosive air near the surface of the iron sample. The synthetic corrosive air containing SO_2_ was produced by roasting (heating in the presence of oxygen) FeS_2_. Reaction rate was controlled by monitoring the temperature and replenishing the reactant as deemed necessary. The SO_2_ generated was pumped into the reaction chamber. The humidified air was generated by passing dry air through a heated MQ water cooled by a circulation tap water. The dry air had a flux of 1.3 l min^−1^, corresponding to a velocity of 3.5 cm·s^−1^ over the sample, which is in the lower range of normal indoor air-flow conditions [33]. The experiments were followed for 60 days and ex situ FAAS measurements and speciation analysis on the iron samples were made after exposure in the chamber. Similar procedure of digestion as described above was used for sample preparation for the FAAS and speciation analysis.

### 2.7. Method Validation

To verify the accuracy and precision (repeatability) of the method, recovery study was carried out using standard solutions spiked on triplicates of the corrugated iron. The good recovery values obtained confirmed the accurate determination of Cr(VI) and total chromium by UV-Vis and FAAS, respectively. At 95% confidence level, the range of % recovery was found to be 93.00 ± 3.09 to 105 ± 4.27 with *t* = 4.30 and degree of freedom 2 for *n* = 3.

### 2.8. Statistical Analysis

Statistical significance of the differences in the levels of Cr detected in duplicate samples of different types or sites (with reference to tannery effluent outlet point) was evaluated using one-way ANOVA at *P* = 0.05. All statistical analyses were performed using SAS Version 9.1.

## 3. Results and Discussions

### 3.1. Determination of Chromium in Corrugated Iron

**Figure 1:**
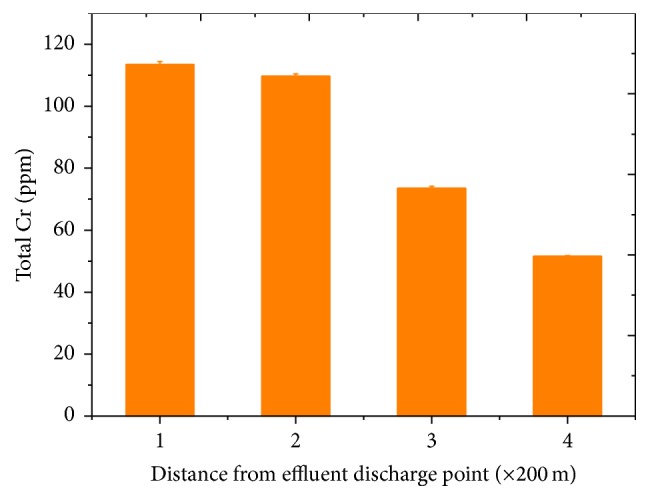
The levels of total Cr(III) corrected for blank (new material) detected in exposed samples against distance from the tannery.

Chromium is naturally present in corrugated iron roof as component of the steel alloy. But this natural level is expected to increase with Fe coupled atmospheric transformation of the hexavalent Cr into the trivalent Cr in areas where industrial activities can lead to atmospheric pollution by Cr(VI). We determined total Cr levels as well as the levels of major species (Cr(III) and Cr(VI)) from speciation experiments in three different groups of the roofing material. The levels of total Cr corrected for blank (new material) detected in exposed samples against distance from the tannery are presented in Figure 1. The levels of total Cr detected in the damaged materials ranged from 113.892 ± 0.17 ppm to 53.05 ± 0.243 ppm. The levels showed a decreasing trend with distance from the tannery. The mean concentrations of Cr in new corrugated iron samples purchased from four shops and that of four control samples collected at 100, 200, 300, and 400 km distances farther from the tannery were 8.5 ± 0.045 and 9.66 ± 0.704 ppm, respectively. The maximum levels of Cr detected in new and control samples were 8.908 and 10.433 ppm, respectively, which is still lower than the minimum level detected in exposed and damaged samples. No significant differences (*P* = 0.05) among the levels of Cr in control samples collected at different distances (100 km and above) were detected. Similarly the levels of Cr in the new corrugated iron materials purchased from four shops did not show significant (*P* = 0.05) differences.

A speciation experiment showed a similar trend in the levels of Cr(III) and Cr(VI) but with low levels of the latter as compared to the former. The highest concentration of Cr(III) and Cr(VI) was 111.876 ± 0.17 and 2.453 ± 1.54 ppm, respectively, in samples collected at industrial sites.

### 3.2. Directionality of Cr Pollution against Effluent Stream

**Figure 2:**
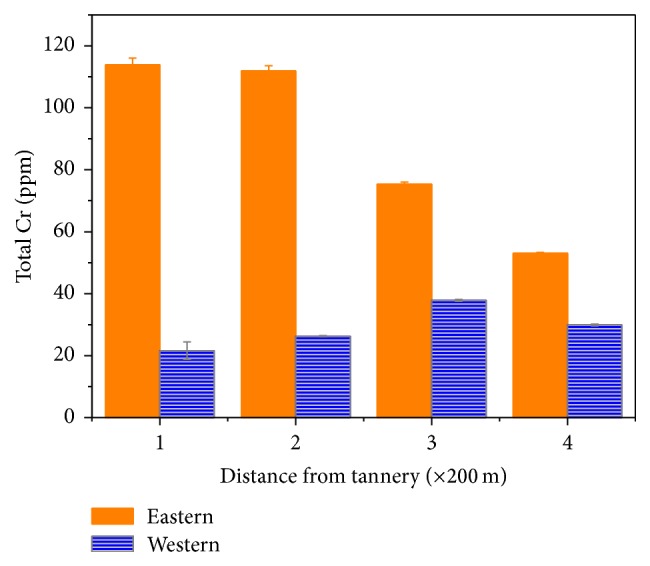
Directional dependence of Cr deposited on the roofing material.

Interestingly, the profile of chromium deposits in the corrugated roofing iron samples showed variation with the direction of the effluent discharge. The concentration was highest in samples collected at the direction of effluent discharge (eastern direction). The results of directional dependence of Cr deposited on the roofing material are shown in Figure 2. Both sets of results reinforce the proposal that the Cr(VI) that might be present in the atmosphere of industrial areas as a result of dislocation or direct emission can transform into the less mobile and insoluble Cr(III) form to increase the load of the latter.

**Figure 3:**
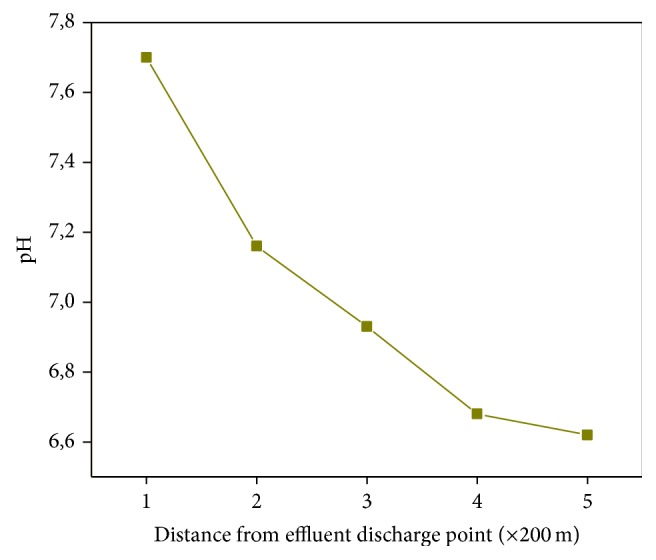
The pH of the corroded corrugated iron materials collected at different distances from the tannery discharge point.

From Figures 2 and 3 it can be summarized that the level of Cr deposits increases in the order: new corrugated, control corrugated, west corrugated, and east corrugated. The increased load of Cr on the iron samples collected at areas in the vicinity of the tanning industry suggests a possible presence of Cr(VI) in the atmosphere that can undergo a coupled transformation to Cr(III). The latter can also settle on the iron materials through direct emission. But considering the mobility and solubility behavior of Cr(III) the former suggestion seems sounder than the latter. The results of laboratory exposure studies performed to verify the above findings and proposed explanations are presented in the subsequent sections.

### 3.3. pH Characterization of Degraded Corrugated Iron Samples

pH is a very important factor in the environmental transformation of chemicals. And also the pH of industrial atmospheres particularly those of tanning industries is expected to be different from that of other areas due to emissions of acid precursor gases (H_2_S and SO_2_). The pH of environmental samples, the corrugated iron roof materials in this case, collected at areas in the vicinity of tanning industries thus needs to be determined as indicator of the corrosivity of the atmosphere. The pH of the degraded corrugated iron materials collected at different distances from the tannery discharge point is shown in Figure 3. An increasing trend in the pH of the samples with distance from the tannery can be observed. This finding is consistent with the fact that proximity to anthropogenic activities determines the level of environmental pollution. In other words, the environmental samples get more and more acidic as they get closer and closer to the industries, especially the tanning industry. The increase in pH with distance might be due to exposure of the water to the atmosphere which can lead to dissolution of SO_2_ in the atmosphere. The pH values did not fall below 6 which is consistent with the finding that the dominant form of Cr in the samples was the basic trivalent form.

### 3.4. A Simulated Laboratory Exposure Study

**Figure 4:**
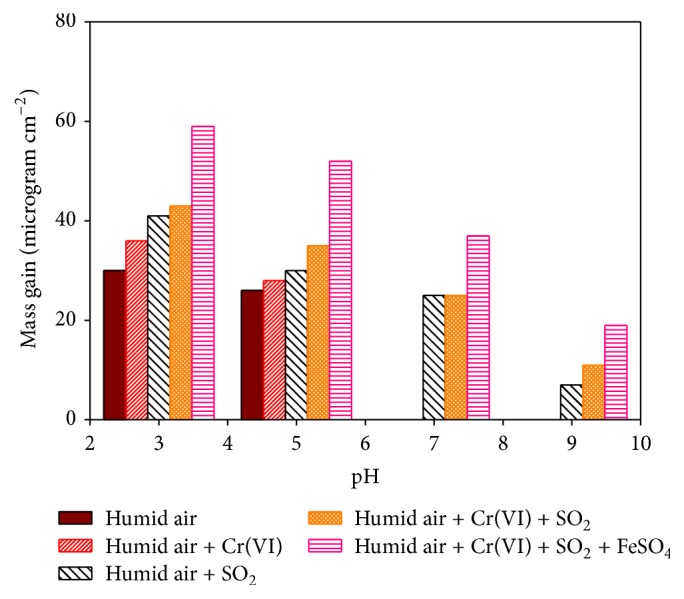
Total mass gain (*μ*g cm^−2^) and corresponding conditions as a function of pH after 60 days of exposure.

In order to study the transformation of Cr(VI) to Cr(III) and the accelerated corrosion of the iron material in the coupled galvanic process, a simulated laboratory exposure experiment on a newly manufactured corrugated iron sample was conducted under varied conditions. Following the exposure, mass gain [34] due to the formation of corrosion products was gravimetrically determined to compare corrosion rates. The level of total chromium was determined by FAAS and speciation into Cr(III) and Cr(VI) was carried out by first determining the hexavalent Cr through UV-Vis method and then calculating Cr(III) from the difference. The increment of Cr(III) was used as indicator of the reduction of Cr(VI) to Cr(III). Five groups of new corrugated iron samples exposed to the simulated conditions of pH 3, 5, 7, and 9 were considered. Group 1 samples consisted of samples just immersed in aqueous solutions of different pH, dried at ambient, and exposed to humid air. The second group consisted of samples immersed in aqueous solutions of Cr(VI) under pH of 3, 5, 7, and 9 and exposed to humid air. The third and fourth groups consisted of samples exposed to synthetic corrosive air containing SO_2_ in absence and presence of Cr(VI), respectively. All other conditions remained the same as that of group 2. Group 5 consisted of samples exposed to synthetic corrosive air containing SO_2_ in the presence of Cr(VI) and FeSO_4_. The sample was immersed in equimolar solution of Cr(VI) and FeSO_4_ and dried at ambient prior to exposure to the corrosive air containing SO_2_. The solution was prepared by dissolving predetermined amounts of FeSO_4_ dissolved in a 50 mL solution of 0.1 M K_2_Cr_2_O_7_ to form the same molar ratio. The results of laboratory exposure studies are shown in Figure 4. All the samples had the exposure times of up to 60 days.

#### 3.4.1. Visual Observation

Visual monitoring of corrosion rate was performed based on the formation of thin layer of light brown to dark red brown rust on the surface of the exposed materials. The rate was recorded in terms of days required to observe the rust formation under different conditions. The visual observation was recorded every 12 hours starting from the first day of the exposure. After one week, visual observation showed that corrosion took place over some of the specimens that are exposed to corrosive air or acidic pH and the colour of the corrosion products was noted. A summary of the number of days required for the formation of the rust during the visual observation is presented in Table 1.

#### 3.4.2. Mass Gain due to Deposition of Corrosion Products

After the immersion experiment, all samples were dried at 55–60°C and weighed before exposure to the corrosive air. The masses of the samples were then determined after 60 days of exposure followed by drying at 55–60°C for 5 minutes. The results of the laboratory exposure studies (total mass gain (*μ*g cm^−2^) and corresponding conditions as a function of pH) are shown in Figure 4. The corrosivity which also paralleled the level of Cr deposits was found to decrease in the order Fe^+2^ + Fe^0^ + SO_2_ + Cr(VI) + humid air, Fe^0^ + SO_2_ + Cr(VI) + humid air, Fe^0^ + SO_2_ + humid air, Fe^0^ + Cr(VI) + humid air, and Fe^0^ + humid air. This was explained by a synergistic effect of SO_2_ and Cr(VI) as polluting species. Further enhancement of the corrosion rate in the presence of ferrous ion is consistent with the fact that Fe^+2^ can undergo a thermodynamic (galvanic) coupling to the redox transformation of Cr(VI) to Cr(III) [35]:

(2) 3Fe2+⟶3Fe3++3e−Cr2O72−+14H++6e−⟶2Cr3+7H2OabCr2O72−+14H++6Fe2+⟶2Cr3++6Fe3++7H2O

For instance, ferrous ion has been used to remove Cr(VI) through reduction to Cr(III) followed by precipitation [6]. Several other reports also showed the use of zerovalent iron to precipitate Cr(III) by reducing the toxic Cr(VI) [4, 7].

We also carried out analysis of the levels of Cr(III) on the samples before and after 60 days of exposure to the corrosive air using FAAS as presented in Table 2. A gradual increment in the levels of Cr(III) has been detected on the samples exposed to Cr(VI) under corrosive conditions. The results are consistent with the proposed coupled transformation of Cr(VI) to Cr(III). This is further verified by the hardly any change recorded with samples exposing the corrosive air in absence of Cr(VI). The data presented in Figure 4 and Tables 1 and 2 reinforce the importance of pH as a key factor in accelerated corrosion of the roofing iron materials involving polluting species.

#### 3.4.3. Impact of Local Surface Stress Cracking on Corrosion Rate

**Figure 5:**
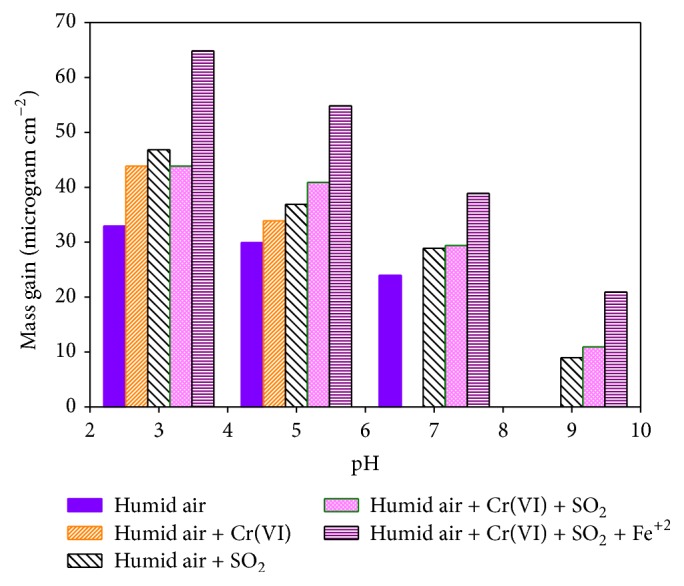
Total mass gain (*μ*g cm^−2^) and corresponding conditions as a function of pH of the scratched iron roof materials after 60 days of exposure.

Stress cracks and physical scratches can play a significant role in the corrosion behavior of materials [36]. When the outer protective layer of a surface is cracked or scratched, the inner layer of the material is exposed to the atmosphere. Such cracks or scratches can occur during manufacturing, transportation, or service not only by chemical means but also by friction with local materials including particulate matters, trees, and its falling parts. The latter is particularly important as it is very common to observe big shade trees near buildings and houses in Ethiopia. Thus, we considered exposure of new but scratched iron materials to corrosive synthetic air under simulated atmospheric conditions and compared their corrosion rates with those without any surface scratches or cracks. Similar five groups of sample materials described in the above sections were considered except, in this case, the surface which is physically scratched with stainless steel before being subjected to the laboratory exposure. The results of the laboratory exposure of scratched iron materials and control samples to synthetic corrosive air under different atmospheric conditions are shown in Figure 5. Comparing the results of Figures 5 and 4, a significant effect of surface scratches on the corrosion rate was observed with all other effects appearing as presented in Figure 4. Our results point to the importance of avoiding surface scratches during manufacturing, packaging, and transporting of corrugated iron roof materials.

## 4. Conclusions

The level of Cr deposits on the samples has been spectrophotometrically determined as indicator of atmospheric Cr pollution leading to accelerated corrosion of the material in tanning industry zones. The experiments showed increasing level of Cr deposits on samples collected from industrial areas as compared to that of the alloy composition of newly manufactured material or control samples collected from presumably nonindustrial areas. The levels increased as sampling sites get closer to the tannery and in the direction of tannery effluent stream. A speciation experiment showed a similar trend in the levels of Cr(III) and Cr(VI) but with low levels of the latter as compared to the former. The observed results can be attributed to atmospheric Cr pollution of tanning industrial areas playing an important role in accelerated corrosion of the materials via a coupled redox process where Cr(VI) is reduced to a stable, immobile, and insoluble Cr(III) deposited on the material and accompanying corrosion of the underlying material. The findings were further verified through laboratory exposure of a newly manufactured material to a simulated synthetic corrosive air containing SO_2_ in the presence and absence of Cr(VI) as polluting species. A relatively faster corrosion rate was recorded in the presence of Cr(VI). Interestingly, a further enhancement in the corrosion rate was recorded in the presence of Fe^+2^ pointing to a synergistic role that Fe^+2^ can play as product of initial corrosion. This study also showed increased corrosion rate when the material is scratched or stress cracked. In conclusion, our results showed possible atmospheric Cr pollution of tanning industrial areas that can play important role in accelerated corrosion of corrugated iron roofs in the areas. The results also suggested the importance of avoiding surface scratches or stress cracks during corrugation, packaging, transporting, and construction or nailing.

## Figures and Tables

**Table 1:** Number of days required for the formation of corrosion products (rust) under different laboratory exposure conditions.

Experiment group	Exposure conditions	Number of days to rust			
		pH			
		3	5	7	9
1	Humid air	15	19	NO	NO
2	Humid air + Cr(VI)	11	15	35	NO
3	Humid air + SO_2_	9	13	25	31
4	Humid air + Cr(VI) + SO_2_	9	12	21	31
5	Humid air + Cr(VI) + SO_2_ + Fe^+2^	7	15	20	17

NO: not observed in 60 days.

**Table 2:** Deposition of Cr(III) as a result of coupled Cr(VI) transformation.

Experiment group	Exposure conditions	Cr(III) (ppm)			
		pH			
		3	5	7	9
1	Humid air	8.9 ± 0.45	8.5 ± 0.045	8.7 ± 0.5	8.12 ± 0.045
2	Humid air + Cr(VI)	11.5 ± 0.08	11.05 ± 0.072	8.9 ± 0.47	8.5 ± 0.2
3	Humid air + SO_2_	8.35 ± 0.55	8.5 ± 0.019	8.7 ± 0.20	8.7 ± 0.008
4	Humid air + Cr(VI) + SO_2_	19.5 ± 0.045	19.02 ± 0.064	10.02 ± 0.035	9.07 ± 0.045
5	Humid air + Cr(VI) + SO_2_ + Fe^+2^	21.5 ± 0.56	19.5 ± 0.53	15 ± 1.1	11 ± 0.78

NO: not observed in 60 days.
